# A Rare Case of Factor XIII Deficiency in the Setting of Cancer Immunotherapy

**DOI:** 10.7759/cureus.15299

**Published:** 2021-05-28

**Authors:** Khashayar Farzam

**Affiliations:** 1 Family Medicine, University of Iowa Hospitals and Clinics, Iowa City, USA

**Keywords:** factor xiii deficiency, mechanical thrombectomy, pembrolizumab, pulmonary embolism, factor xiii, bleeding disorder, massive pulmonary embolism

## Abstract

Factor XIII deficiency is a rare bleeding disorder, which may be congenital or acquired, and is most commonly diagnosed in early childhood. It has a prevalence that is as low as one in 5,000,000. Acquired factor XIII deficiency is considered to be a more rare form, with less than 100 cases reported in the literature. This disorder can be clinically characterized by recurrent and severe unexplained bleeding. This case describes a patient with no prior bleeding disorders, who suffered from recurrent bleeding episodes while being treated with cancer immunotherapy, specifically pembrolizumab, and was subsequently diagnosed with factor XIII deficiency. She required cryoprecipitate infusion due to the persistent bleeding and subsequently developed saddle pulmonary embolism. The patient was not a candidate for tissue plasminogen activator (tPA) due to her recurrent bleeding and required mechanical thrombectomy. Given the timeline of symptoms, the patient likely developed acquired factor XIII deficiency due to her cancer immunotherapy.

## Introduction

Coagulation factor XIII deficiency is a rare bleeding disorder characterized by the formation of unstable clots. It is composed of two subunits, A and B, which are activated by thrombin and calcium. In healthy individuals, fibrin undergoes cross-linking and forms a stable hemostatic clot [1]. Those with deficiency of factor XIII will develop defective cross-linking and form unstable clots. This predisposes these patients to recurrent hemorrhage, which is clinically similar to those with hemophilia [2]. This remains a relatively unknown condition due to its low prevalence. As such, there is a lack of evidence-based guidelines to aid the diagnosis and management of this condition [3].

Patients with this bleeding disorder will either have it congenitally or acquire it later in life. Most cases of factor XIII deficiency are congenital and will generally be diagnosed shortly after birth due to symptoms of recurrent unexpected bleeding. It is autosomal recessive and occurs due to potential gene mutations [4]. The true prevalence is unknown, but the general estimate is approximately as low as one in 5,000,000 at the global level. Symptoms have major variability; some only experience minor symptoms such as mild epistaxis, whereas others experience severe symptoms such as intracranial hemorrhage [5]. Initial coagulation laboratory evaluation is generally unremarkable. Most patients will have a normal prothrombin time (PT), partial thromboplastin time (PTT), international normalized ratio (INR), and bleeding time. Treatment generally involves fresh frozen plasma (FFP) or cryoprecipitate given that these contain factor XIII [6]. Other treatments include factor XIII concentrates and recombinant factor XIII, which are sometimes available as treatment options depending on the jurisdiction [7]. In some cases, immunosuppressive therapy may also be warranted for some cases of acquired factor XIII deficiency.

Acquired factor XIII deficiency is the less common form of this bleeding disorder, with a limited number of cases being reported in the literature. It develops due to an autoantibody, which may be triggered due to an autoimmune illness, malignancy, and/or a medication. Like many other illnesses, it can also be idiopathic. Systemic lupus erythematosus (SLE) is one possible trigger for the development of acquired factor XIII deficiency. Some medications such as phenytoin, procainamide, and isoniazid have also been linked to acquired factor XIII deficiency [8]. The true scope of the etiologies remains unknown given that a patient’s bleeding symptoms may not be severe enough to warrant comprehensive investigation and may go undiagnosed. Patients are also older when they develop this acquired factor deficiency, and clinical suspicion for any congenital etiologies may be low. Cancer immunotherapy, specifically pembrolizumab, has not shown a previous link to factor XIII. Based on what is known in the current literature, this is the first case of factor XIII deficiency that was likely acquired due to pembrolizumab therapy.

## Case presentation

The patient is a 71-year-old female who was undergoing treatment with pembrolizumab for metastatic vulvar melanoma. Her past medical and surgical history was relatively uncomplicated and non-pertinent. She had no personal history of any bleeding disorders or symptoms and no family history of bleeding disorders.

She was diagnosed with malignant vulvar melanoma and underwent radical vulvectomy and radiation therapy. Three months later, she also began treatment with pembrolizumab. The patient was enrolled in an RP1 clinical trial several months later, where a genetically modified herpes simplex 1 (HSV1) virus was injected into the tumor to generate an anti-tumor response. She was noted to have significant bruising diffusely around her body and experienced significant bleeding following her RP1 injections. Notably, the patient expressed having persistent bruising and significant bleeding symptoms shortly after beginning the pembrolizumab treatment. PT and PTT were obtained, which were 11 and 25 seconds, respectively, which were within normal limits. Her INR was 1.0. In addition, platelet function studies were performed and were normal. The decision was made to obtain a factor XIII level.

The patient’s factor XIII level was 42%, well below the accepted laboratory cut-off of 69%. She continued to experience significant bleeding following the intratumor injections, in addition to bleeding from her perineum following bowel movements. This prompted us to give her 1 unit of cryoprecipitate on outpatient follow-up. Further history taking from the patient revealed that she did not have any bleeding-related symptoms prior to the initiation of pembrolizumab therapy.

The patient presented to the emergency room two days after her cryoprecipitate infusion with the complaint of shortness of breath. Her blood pressure was approximately 70/40 mm Hg and she was tachycardic. She underwent a computed tomography (CT) angiography of her chest, which revealed a saddle embolism with evidence of right heart strain. She was subsequently started on a heparin infusion along with a norepinephrine infusion and was transferred to the medical intensive care unit (ICU). Since the patient was hypotensive in the setting of a saddle embolism, her treatment options included tissue plasminogen activator (tPA) or mechanical thrombectomy. Given her factor XIII deficiency, it was determined that she was not a candidate for systemic or catheter-guided tPA therapy due to the significant bleeding risks.

**Figure 1:**
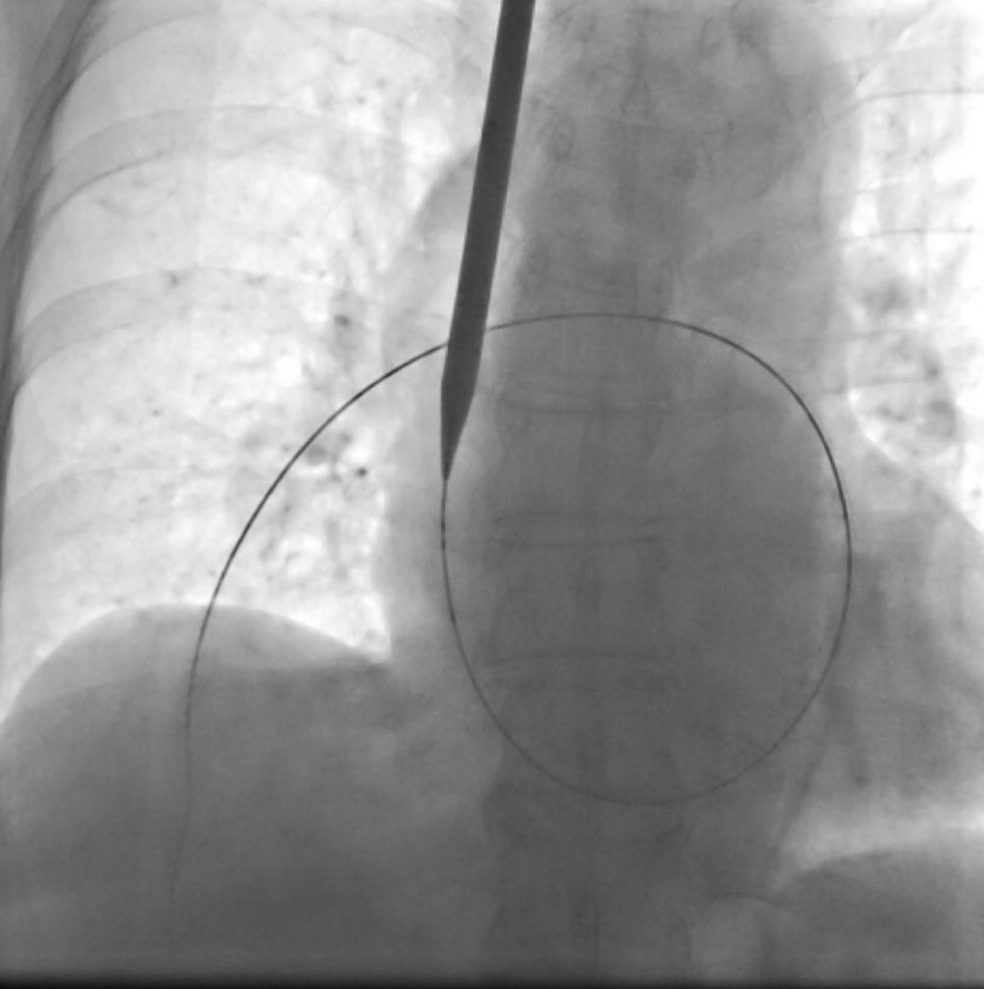
Right Heart Catheterization

The patient underwent further workup prior to definitive therapy. A transthoracic echocardiogram was obtained, which revealed a tricuspid annular plane systolic excursion of 15 mm and evidence of decreased right ventricular systolic function. Thromboelastography (TEG) was obtained, which revealed an initial clot formation of 10.8 minutes (elevated), kinetics of 2.8 minutes (normal), clot angle of 55.6 degrees (normal), maximum amplitude of 64.7 mm (normal), clot strength G of 9.2 Kd/cm^2^ (normal), and coagulation index of -3.7 (decreased). Factor VIII levels were also highly elevated at 409% with the upper limit of normal being 200%. It was possible that the cryoprecipitate infusion may have been the cause of her saddle embolism given that it can elevate factor VIII levels. However, the patient’s metastatic malignancy would also have placed her in a coagulopathic state. Fortunately, the patient underwent successful right heart catheterization and mechanical thrombectomy and was transferred out of the ICU the next day (Figure 1). She was evaluated several weeks later on outpatient follow-up, after the pembrolizumab had been discontinued, and her factor XIII level had normalized.

.

## Discussion

Factor XIII deficiency is a rare bleeding disorder. It can induce a broad range of symptoms that vary from mild bleeding to severe hemorrhagic episodes. Most often, factor XIII deficiency is congenital and not acquired later in life. This patient’s age and timeline of symptoms directly correlated with the use of pembrolizumab, which makes it likely that this factor deficiency was acquired. The strongest evidence favoring her condition being induced by cancer immunotherapy would be the normalization of her factor XIII level after discontinuation of the pembrolizumab. She also had no prior bleeding-related symptoms prior to the initiation of pembrolizumab and had no other family history of bleeding disorders. Shortly after initiating cancer immunotherapy, she began to experience recurrent unexpected bleeding and bruising. The patient also had no other symptoms of other autoimmune illness and had not initiated any other new medications, all of which could trigger acquired factor XIII deficiency. It is possible that her malignancy was the etiology of the acquired factor XIII deficiency; however, she had developed the cancer much earlier than when she began pembrolizumab therapy, and the level normalized after the likely offending agent was discontinued. Based on current available literature review, this is the first documented case of likely acquired factor XIII that is due to pembrolizumab and cancer immunotherapy.

TEG was performed shortly after the patient’s admission to the ICU. However, its diagnostic and prognostic utility is relatively unknown for a patient presenting in such a specific clinical scenario. It also lacks standardization across institutions. There is some evidence that TEG may be more sensitive than solubility tests for detecting factor XIII deficiency [9], which could potentially make it a useful diagnostic tool in the future.

The patient required treatment for her recurrent bleeding and received cryoprecipitate infusions. It is possible that this may have been a provoking factor in the development of her venous thrombosis and subsequent saddle embolism, especially with the highly elevated factor VIII levels. As such, physicians should be mindful of the risks associated with cryoprecipitate or FFP infusion when a patient has other coagulopathic risk factors. It is not an unusual clinical task to balance the risk of bleeding and thrombosis. Knowledge of any bleeding disorders or thrombophilias can help assist clinical decision-making and risk mitigation.

## Conclusions

Physicians should be aware of the possibility of acquired factor XIII deficiency when dealing with unexplained bleeding in a patient undergoing pembrolizumab therapy or other cancer immunotherapy. In addition, therapeutic treatments for factor XIII deficiency may predispose the patient to the development of venous thrombosis. It would be reasonable to pursue mechanical management rather than thrombolytic therapy in these patients due to the high risk of bleeding.
